# A simultaneous diagnosis and genotyping method for global surveillance of cetacean morbillivirus

**DOI:** 10.1038/srep30625

**Published:** 2016-08-03

**Authors:** Wei-Cheng Yang, Bi-Jhen Wu, Eva Sierra, Antonio Fernandez, Kátia R. Groch, José Luiz Catão-Dias, Kristi West, Kun-Wei Chan

**Affiliations:** 1 National Chiayi University, Chiayi, Taiwan; 2 University of Las Palmas de Gran Canaria, Las Palmas de Gran Canaria, Canary Islands, Spain; 3 University of São Paulo, São Paulo, Brazil; 4 Instituto Baleia Jubarte (Humpback Whale Institute), Caravelas, Brazil; 5 Hawaii Pacific University, Kaneohe, Hawaii, USA

## Abstract

Cetacean morbillivirus (CeMV) is considered one of the most important viral pathogens in cetaceans. CeMV outbreaks of lethal disease have repeatedly been observed in Europe, the Americas, and Australia, while large herds of gregarious species were found to be the likely reservoirs and sources of CeMV infection to susceptible species in the Atlantic and Pacific Oceans. Furthermore, three new strains were detected recently in Hawaii, Brazil and Australia. To clarify the real global distribution of CeMV and possible carriers, we showed a novel technique successfully diagnosing and distinguishing different virus strains (DMV, PWMV and novel CeMVs) using FFPE samples from 1996 to 2011. This efficient method that combines qRT-PCR and high resolution melting (HRM) could be applied to the future retrospective global studies for better understanding of different prevalence and outbreak conditions among ocean basins and the mechanism of variable host response to pathogens.

Cetacean morbillivirus (CeMV) is considered one of the most important viral pathogens in cetaceans^1^. It includes three well-characterized strains (porpoise morbillivirus, PMV; dophin morbillivirus, DMV; pilot whale morbillivirus, PWMV) and three new strains from Hawaii, Brazil, and Australia detected by RT-PCR^2^. In infected cetaceans, the virus causes serious respiratory, central nervous system disease and immuosuppression that lead to serious secondary bacterial and fungal infections^3^,^4^. Outbreaks of lethal CeMV infection have recurrently been noticed in Europe, the Americas, and Australia since the late 1980s^2^. It is intriguing that some cetaceans are positive for CeMV by RT-PCR without typical pathological findings resulting from CeMV infection^5^,^6^,^7^,^8^, indicating possible subclinical CeMV infection in certain species and areas. For greater understanding of the role of potential carriers and would clarify the global distribution of this important pathogen, a reliable method for conducting retrospective survey of CeMV is essential. As compared to ultracold frozen tissues, formalin-fixed paraffin-embedded (FFPE) samples that represent recent and historical stranding events are more widely available for retrospective survey. However, the regular RT-PCR method for CeMV detection^9^ is not suitable for FFPE samples because formaldehyde creates cross-links between nucleic acids and proteins resulting in short amplicons (<200 bp). Krafft *et al*.^10^ designed a protocol using RT-PCR with Southern blot that allows CeMV RNA amplification in FFPE samples. However, this method is laborious and time-consuming, particularly when a large number of samples need to be tested. We describe a novel scheme that combines real-time RT-PCR (qRT-PCR) and high resolution melting assay (HRM) for rapid genotyping using FFPE samples stemming from 1996 to 2011 that efficiently detects and differentiates the global spectrum of CeMV strains.

## Results and Discussion

**Figure 1:**
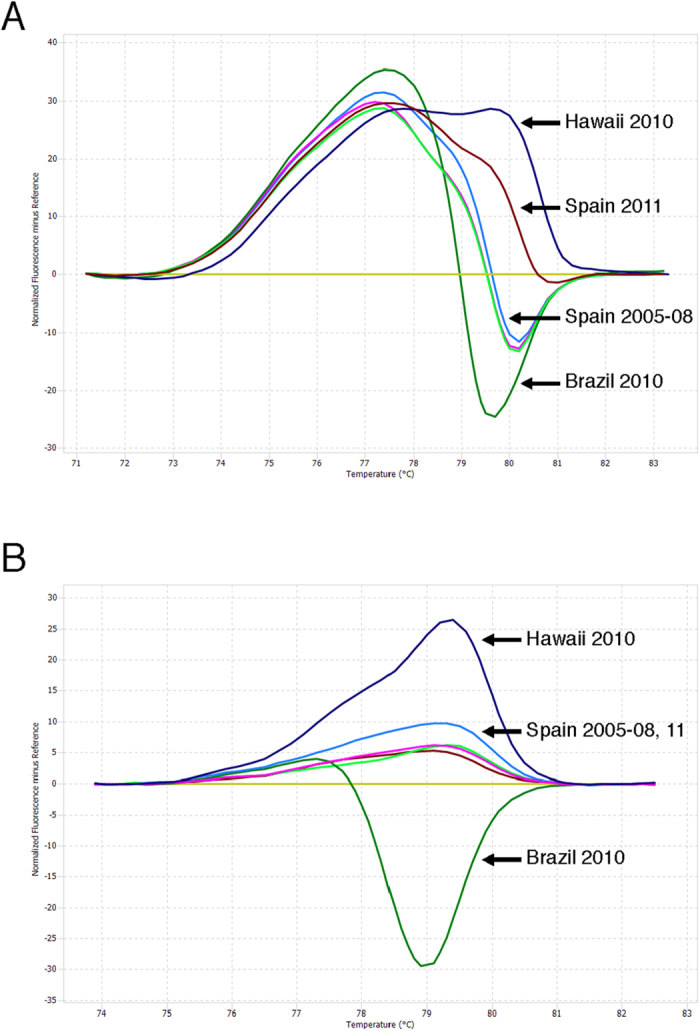
Derivative high-resolution melting curves comparing individual cetacean morbillivirus strains. Pilot whale morbillivirus was converted to a horizontal line as reference. (**A**) P1 segment, (**B**) P2 segment.

Two primer sets (P1 and P2) were designed for use with the qRT-PCR to amplify phosphoprotein (P) gene of CeMV. The lower limit of detection of P1 and P2 was both 10^1^ copies/reaction (P1: R^2^ = 0.997, efficiency = 103.37%; P2: R^2^ = 0.988, efficiency = 107.24%). Both primer sets successfully detected different CeMV strains in the 7 cases previously confirmed as CeMV infections^6^,^11^,^12^,^13^,^14^,^15^ while the other 20 samples of other stranded cetaceans in western Pacific collected from 2005 to 2013 were negative. We are confident that high-quality RNA was extracted from all of the FFPE samples since all 27 samples were positive (Cq = 19–29) with the ß-actin primer set. In the difference graph of HRM analysis, the melting profiles of each sample were compared to the pilot whale morbillivirus (PWMV) that was converted to a horizontal line. The HRM profiles with P1 primer set showed five distinct groups: (I) Hawaii 2010 CeMV (II) Spain 2011 DMV (III) Spain 2005, 2007 and 2008 DMV (IV) Brazil 2010 CeMV (V) PWMV (Fig. 1A). The HRM profiles with P2 primer set showed four distinct groups: (I) Hawaii 2010 CeMV (II) Spain 2005, 2007 and 2011 DMV (III) Brazil 2010 CeMV (IV) PWMV (Fig. 1B). Single-nucleotide differences (Table 1) resulting in slight shifts in the melting domain were clearly detected. The melt profiles within one CeMV strain were consistent in all samples and replicates. Table 2 shows the Cq and melt peak temperature (Tm) of the positive samples.

It was proposed that there are two CeMV lineages: CeMV-1 includes DMV, PMV, PWMV, and Hawaii strain; CeMV-2 includes strains from Brazil and Australia^2^. The representative CeMV strains from nearly all regions worldwide were included in this study, and the results provide evidence that the HRM-based method presents a valid and efficient approach to the detection and subtyping of the CeMV P gene of these two lineages. This method does not need specific probes in addition to the specific primers for each morbillivirus strain. A cheap fluorescent molecule (Eva-Green^®^), which is readily available to diagnostic laboratories, was used. By using two primer sets targeting two different sites for increasing the detection ability, and HRM that is able to differentiate sequences differing by one single nucleotide, it is theoretically possible to detect small variations in the P gene among virus strains. Because the decomposition status and formalin fixation time of these samples are varied, ß-actin was used as a control for the loading and integrity of the RNA. The organs tested in this study except the lymph nodes and spleen from the Longman’s beaked whale (Table 2) present typical morbillivirus lesions, including non-purulent encephalitis, interstitial bronchopneumonia, syncytia, eosinophilic inclusion bodies, and lymphoid depletion with germinal center necrosis. It is imperative that the morbilliviral RNA was detected in every FFPE sample with adequate Cq value, representing the high sensitivity of this method. Furthermore, the PWMV sample collected in 1996 can be used for diagnosis, indicating that it is feasible to conduct retrospective study of CeMV infection with this method. Therefore, it can represent a valuable screening method for the initial detection of CeMV strains worldwide, especially for the ocean basins that no mass mortality has been reported and for the stranded cetaceans that show no typical lesions of morbillivirus infection.

The character and existing of subclinical CeMV infection remains conjectural although acute and subacute systemic presentations and chronic CNS infection causing death have been described^2^. The first possible subclinical CeMV infection was reported that morbilliviral RNA was detected in three of the five DMV seropositive common dolphins (*Delphinus delphis*) stranded along the southern California coast from 1995 to 1997 without lesions characteristic of morbilliviral disease^5^. In central Pacific, morbillivirus infection was detected in a juvenile Longman’s beaked whale (*Indopacetus pacificus*) in 2010^6^ and a neonate sperm whale (*Physeter macrocephalus*) in 2011^7^ in Hawaii while typical morbillivirus lesions were not detected in both individuals. In 2013, morbilliviral RNA was detected in brain and lung samples from 22 striped dolphins (*Stenella coeruleoalba*), one bottlenose dolphin (*Tursiops truncatus*) and one fin whale (*Balaenoptera physalus*) stranded along the Italian Tyrrhenian Sea coast during an unusual mortality event^8^. However, the causal factors of this event still needs further investigation because none of the positive individuals had characteristic morbillivirus lesions and other infectious agents were concurrently detected in a high percentage of these individuals. It is not clear whether these cases mentioned above actually represent subclinical infection or an atypical viral strain/host presentation. It is recommended to use the qRT-PCR method in the present study and histology/IHC concurrently to further explore the pathogensis of CeMV infection.

The many advantages of qRT-PCR over conventional RT-PCR include no requirement to pour gels or use hazardous chemicals (e.g. ethidium bromide), PCR product contamination is eliminated by the closed-tube format, and small reaction volumes (10 μl) produce very time- and cost-effective assays. HRM analysis readily detects only one to two nucleotide difference compared to other sequences of CeMV strains. Such variants may not be revealed by the qRT-PCR methods with the TaqMan probe or a hybridization probe. If a novel or unusual CeMV strain identified by HRM analysis, it can be more extensively characterized by direct sequencing or obtaining long PCR amplicon using archived frozen tissues since the amplicons in this study are short (85 bp and 72 bp) and may not be appropriate to perform phylogenetic analysis. In conclusion, HRM of the P gene could represent a sensitive and rapid method for both retrospective studies of long-term stored FFPE samples and for implementing epidemiological surveillance of CeMV. We successfully validated a qRT-PCR with HRM using representative CeMV strains from nearly all regions worldwide. High sensitivity enables reliable detection of this virus in FFPE samples and in low viral load samples. Through investigation of the relationship between virus variation and responsiveness to CeMV in cetaceans, this method could lead to a greater understanding of not only the differing prevalence and outbreak conditions between Atlantic and Pacific oceans, but also the mechanisms of variable host response to pathogens. Future studies based on this method could elucidate the role of virus strains in disease susceptibility, resistance, and progression.

## Methods

**Figure 2:**
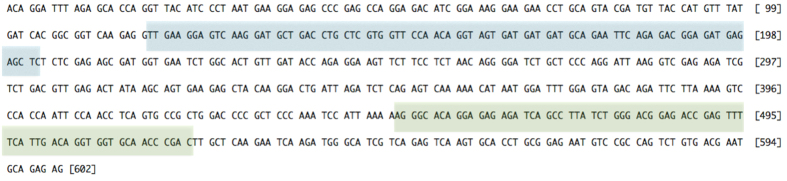
The 602 bp fragments of P gene (GenBank accession number: HQ829973) contain the fragments that are amplified with the primer sets of P1 (blue) and P2 (green) using qRT-PCR.

Primers were selected on the basis of alignments of P gene sequences of CeMV strains from GenBank. The amplification length of two primer sets (P1 and P2) was 85 bp and 72 bp, respectively (Fig. 2)(5′-3′: P1F-TTGAAGGAGTCAAGGATGCTG; P1R-GAGCTCTCATCTCCGTCTCTG; P2F-AGGGCACAGGAGAGAGATCA; P2R-ATTGGGTTGCACCACTTGTC). To assess the sensitivity of the assay, we constructed a plasmid that contains the P gene of CeMV (Fig. 2). The fragment of the P gene inserted into the plasmid was synthesized according to a dolphin morbillvirus (DMV) strain (GenBank accession number: HQ829973). The plasmid was serially diluted 10-fold in sterile water and used to test the assay. The linear range of sensitivity was 10^1^–10^7^ copies. Seven FFPE samples diagnosed with CeMV infection from cetaceans stranded in Spain, Hawaii and Brazil from 1996 to 2011^6^,^11^,^12^,^13^,^14^,^15^ and 20 FFPE samples from lung or lymphoid organs of other stranded cetaceans in western Pacific (Taiwan) collected from 2005 to 2013 were used (Table 2). The fixative of these samples was 10% neutral buffered formalin. These samples were processed according to routine histopathological methods and stained with haematoxylin and eosin for microscopic examination. Total RNA of the FFPE samples were extracted using RNeasy FFPE kit (Qiagen). RNA integrity was monitored routinely using denaturing gel electrophoresis. Total RNA concentration ranged from 5.4 to 8.9 ng/μl in the extracted samples, as measured using fluorescence-based quantitation method (Qubit fluorometer with a Quant-iT RNA Assay Kit, Invitrogen) suggested in Minimum Information for Publication of Quantitative Real-Time PCR Experiments (MIQE) guideline^16^ for RNA quantity and purity determination. ß-actin was used as an internal RNA quality control (β-actinF-AGGACCTCTATGCCAACACG; β-actinR-CCTTCTGCATCCTGTCAGC).

RNA was reverse transcribed in a final volume of 20 μl including 5 μl of QuantiTect reverse-transcription master mix (Qiagen), 0.7 μM of gene-specific primers and 14 μl of RNA. The reaction was performed at 42 °C for 15 min followed by inactivation of the reverse transcriptase by heating at 95 °C for 3 min. The qPCR assay was performed in a 10 μl volume containing 5 μl of IQ2 Fast qPCR System master mix (Biogenesis), 0.4 μM of each primer and 2 μl of diluted cDNA. The qPCR cycling was performed using the Eco Real-Time PCR System (Illumina) as follows: 95 °C for 2 min followed by 45 cycles, each consisting of 95 °C for 5 s, 60 °C for 30 s. After completion of the qPCR step, the amplified target was gradually denatured by increasing the temperature from 55 °C to 95 °C at 0.1 °C/s to produce a characteristic melting profile. Significant deviations from the horizontal line in the difference graph were indicative of sequence changes within the amplicon of the target gene analyzed. To assess reproducibility, every sample was analyzed in triplicate and on three different plates. Tm and the standard deviation (SD) were determined for replicate analyses of each specimen using tools provided in the software. The amplified products were cloned into TA plasmid vector. The nucleotide sequences were determined using Sanger sequencing, and were compared to the P gene sequences of CeMV from GenBank.

## Additional Information

**How to cite this article**: Yang, W.-C. *et al*. A simultaneous diagnosis and genotyping method for global surveillance of cetacean morbillivirus. *Sci. Rep.* **6**, 30625; doi: 10.1038/srep30625 (2016).

## Figures and Tables

**Table 1:** Pairwise comparison of nucleotide substitutions for the cetacean morbillivirus strains included in this study, based on P1 (below diagonal) and P2 (above diagonal) fragment of the phosphoprotein gene.

	Hawaii 2010	Spain 2011	Spain 2005	Spain 2007	Spain 2008	PWMV	Brazil 2010
Hawaii 2010	—	1	1	1	1	5	4
Spain 2011	2	—	0	0	0	6	5
Spain 2005	1	1	—	0	0	6	5
Spain 2007	1	1	0	—	0	6	5
Spain 2008	1	1	0	0	—	6	5
PWMV	2	2	3	3	3	—	5
Brazil 2010	6	6	7	7	7	6	—

**Table 2:** The samples used in this study and positive results.

Species	Years	Countries	Virus	Original diagnosis	Organs tested in this study	P1 Cq ± SD/Tm ± SD	P2 Cq ± SD/Tm ± SD	Literature cited
*Globicephala macrorhynchus*	1996	Canary Islands, Spain	PWMV	RT-PCR	C	29.70 ± 0.16/80.01 ± 0.07	28.37 ± 0.03/79.22 ± 0.07	11
*Tursiops truncatus*	2005	Canary Islands, Spain	DMV	IHC, RT-PCR	LN	22.03 ± 0.14/79.53 ± 0.06	26.05 ± 0.05/79.36 ± 0.07	12
*G. melas*	2007	Spain	DMV	IHC, RT-PCR	C, L, LN, S	21.24 ± 0.38/79.53 ± 0.05	22.99 ± 0.41/79.41 ± 0.03	13
*Stenella coeruleoalba*	2008	Canary Islands, Spain	DMV	IHC, RT-PCR	C	25.7 ± 0.26/79.54 ± 0.06	29.1 ± 0.51/79.41 ± 0.06	14
	2011	Canary Islands, Spain	DMV	IHC, RT-PCR	C	27.96 ± 0.81/80.11 ± 0.04	29.1 ± 0.51/79.40 ± 0.07	14
*Indopacetus pacificus*	2010	Hawaii, USA	CeMV NL	IHC, RT-PCR	LN, S	28.59 ± 0.15/80.40 ± 0.01	30.83 ± 0.36/79.72 ± 0.06	6
*Sotalia guianensis*	2010	Espirito Santo, Brazil	CeMV NL	IHC, RT-PCR	LN	32.53 ± 0.54/78.92 ± 0.09	32.23 ± 0.24/78.51 ± 0.08	15
20 species*	2005–2013	Taiwan	Negative		L, LN, S	NA	NA	This study

Abbreviations are: PWMV = pilot whale morbillivirus, DMV = dolphin morbillivirus, CeMV = cetacean morbillivirus, NL = new lineage of CeMV, C = cerebrum, L = lung, LN = lymph node, S = spleen, Cq = quantification cycle, Tm = melt peak temperature, SD = standard deviation. ^*^Balaenoptera acutorostrata, B. omurai, Physeter macrocephalus, Kogia breviceps, K. sima, Neophocaena phocaenoides, Steno bredanensis, Sousa chinensis, Grampus griseus, Tursiops truncatus, Stenella attenuata, S. longirostris, S. coeruleoalba, Lagenodelphis hosei, Peponocephala electra, Feresa attenuata, Pseudorca crassidens, Globicephala macrorhynchus, Ziphius cavirostris, Mesoplodon densirostris.
